# Review on Some Confusion Produced by the Bicontinuous Microemulsion Terminology and Its Domains Microcurvature: A Simple Spatiotemporal Model at Optimum Formulation of Surfactant-Oil-Water Systems

**DOI:** 10.1021/acsomega.3c00547

**Published:** 2023-03-02

**Authors:** Jean-Louis Salager, Ronald Marquez, Miguel Rondón, Johnny Bullón, Alain Graciaa

**Affiliations:** † Laboratorio FIRP, Universidad de Los Andes, Mérida 5101, Venezuela; ‡ Universidad Industrial de Santander, Bucaramanga 680002, Colombia; ⊥ ICP Ecopetrol, Piedecuesta 681011, Colombia; § Université de Pau et Pays de l’Adour, UMR 5150 TOTAL-CNRS-UPPA, BP 1155, Pau 64013 Cedex, France

## Abstract

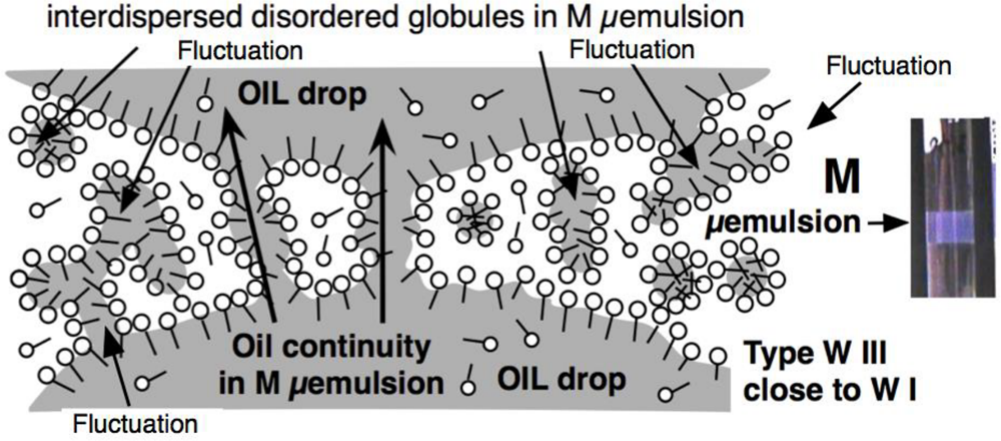

Fundamental studies have improved understanding of molecular-level properties and behavior in surfactant-oil-water (SOW) systems at equilibrium and under nonequilibrium conditions. However, confusion persists regarding the terms “microemulsion” and “curvature” in these systems. Microemulsion refers to a single-phase system that does not contain distinct oil or water droplets but at least four different structures with globular domains of nanometer size and sometimes arbitrary shape. The significance of “curvature” in such systems is unclear. At high surfactant concentrations (typically 30 wt % or more), a single phase zone has been identified in which complex molecular arrangements may result in light scattering. As surfactant concentration decreases, the single phase is referred to as a bicontinuous microemulsion, known as the middle phase in a Winsor III triphasic system. Its structure has been described as involving simple or multiple surfactant films surrounding more or less elongated excess oil and water phase globules. In cases where the system separates into two or three phases, known as Winsor I or II systems, one of the phases, containing most of the surfactant, is also confusedly referred to as the microemulsion. In this surfactant-rich phase, the only curved objects are micellar size structures that are soluble in the system and have no real interface but rather exchange surfactant molecules with the external liquid phase at an ultrafast pace. The use of the term “curvature” in the context of these complex microemulsion systems is confusing, particularly when applied to merged nanometer-size globular or percolating domains. In this work, we discuss the terms “microemulsion” and “curvature”, and the most simple four-dimensional spatiotemporal model is proposed concerning SOW equilibrated systems near the optimum formulation. This model explains the motion of surfactant molecules due to Brownian movement, which is a quick and arbitrary thermal fluctuation, and limited to a short distance. The resulting observation and behavior will be an average in time and in space, leading to a permanent change in the local microcurvature of the aggregate, thus changing the average from micelle-like to inverse micelle-like order over an extremely short time. The term “microcurvature” is used to explain the small variations of globule size and indicates a close-to-zero mean curvature of the surfactant-containing film surface shape.

## Introduction

The present article discusses the 50 years of evolution in the handling of the formulation of surfactant-oil-water (SOW) systems currently used in many different applications such as detergency and other cleaning processes, foods, paints, petroleum production, emulsion stabilization and breaking, agrochemicals, pharmaceuticals, cosmetics, and even explosives.^1^ The formulation of SOW systems involves variables that influence the equilibrium properties, such as phase behavior, adsorption, solubility and solubilization, surface and interfacial tensions, and wettability.^2^ It also involves the effects of these systems on nonequilibrium heterogeneous mixtures, including various types of dispersions, particularly liquid–liquid systems like macro-, mini-, micro-, and nanoemulsions, which are of practical interest due to their stability, drop size, and rheological properties.^3−6^

The use of SOW systems can be traced back to ancient Sumerian civilization, where cuneiform records reveal their application in the cleaning of wool through the removal of fat, a process referred to currently as detergency. The science of soap-making has evolved over the past 4000 years through trial and error, eventually leading to a better understanding of the molecular mechanisms behind cleaning at interfaces. The study of surface and interface science, including the physical and chemical aspects of adsorption, wetting, adhesion, friction, and mono- and bilayer films, has involved the investigation of intermolecular forces in colloidal dispersions by renowned scientists such as van der Waals, Keesom, London, Langmuir, Debye, Huckel, Boltzmann, Derjaguin, Landau, Vervey, Overbeek, Miller, Hirasaki, and others. Physicists including Einstein and Smoluchowski also made important contributions predicting Brownian motion through the study of molecular fluctuations, which was later confirmed experimentally by Perrin, who received a Nobel Prize for his work. These fundamental studies have improved our understanding of the forces at play at the molecular level and have led to the development of the field of colloid and interface science, which aims to provide a theoretical framework for practical applications.

In the early 1900s, the introduction of new amphiphilic molecules with polar groups other than those found in soaps, such as ionic sulfate and sulfonate groups and nonionic polyethoxylated chains, expanded the surfactant-based cleaning formulations into a wide range of applications. These molecules, which were less sensitive to precipitation by polyvalent cations commonly found in washing water, were no longer referred by their tension-reduction properties, like “tensioactif” in French and “tenside” in German but were definitively renamed surface active agents (surfactants) in 1950. This happened in industrial research centers, particularly in the United States who started to publish not only knowledge but also some know-how information, often in relation with commercial products. Over the following half century, there was significant growth in the surfactant business with a divulgation increase through conferences on specific topics, particularly enhanced oil recovery (EOR).

This expansion led to the publication, starting in the late 1970s, of over 200 books and encyclopedias and thousands of articles with the contribution of numerous authors, not only from the academy but also from industry. With very few exceptions like the sometimes called “surfactant science bible” written by Milton Rosen first in 1978 with 300 pages, and now in its fourth edition with a doubled size,^6^ most of the review books have been written by multiple authors, resulting in discrepancies between different sources and even between chapters within the same book. One of these discrepancies is around the term “microemulsion” and the multiple meanings of the word “curvature”.^7−12^ As stated by Rosen in his courses^13^ and in the preface of his exceptional book on practical applications of surfactants,^14^ these disagreements and inconsistencies are probably due to the strong differences existing between theoretical knowledge and practical know-how. Rosen’s comments essentially mentioned that this situation has reached the point where nobody can be a specialist in both approaches at the same time.

The main issue is that since the 1990s the amount of available information in both types of approaches became excessive, as shown in a recent review,^1^ making it impractical to review and assimilate all the available information in less than three years of full-time study. Consequently, only a partial formation could be carried out for individuals who are going to be involved with the use or formulation of SOW systems, such as tutorial activities with a limited time like 2–3 courses with 50–60 h per year. This presents a challenge for effectively teaching individuals about surfactants, colloids, and interfaces, and many researchers in these fields are likely unaware of important issues due to this information overload. This has practical consequences for the field as a whole.

There are over 50 books with the term “microemulsion” in the title, over 150 books that have at least one chapter with this term in the title, and more than 500 journal articles that use this term. However, many of these publications are limited to theoretical concepts or applications and contain misleading or nongeneralized comments, as well as repeated confusion. The following list of ten works represents a carefully selected bibliography for readers of this review to understand the challenges surrounding the nomenclature and meanings of microemulsion mesophase structures and curvature. Only three of these were written by authors in a very consistent way like the fundamental and practical basics in “surfactant science and applications” by M. J. Rosen,^6^ the “microemulsion formulation review” by M. Bourrel and R. S. Schechter,^15^ and the very recent tutorial screening on “emulsions, microemulsions and foams” by D. Langevin,^3^ who included both words emulsion and microemulsion in the title, thus clearly indicating that they have different meanings. The other selected books on “microemulsions” in their title were edited by notable contributors like L. Prince,^16^ K. L. Mittal,^17^ M. Fanun,^18^ P. Kumar and K. L. Mittal,^19^ C. Stubenrauch,^20^ L. E. Scriven and K. L. Mittal,^21^ R. Zana,^22^ and J. N. Israelachvili.^23^

These selected references include the most influential opinions and some controversies on the topic of microemulsion structures and curvature meanings in the last 50 years from the most productive researchers on the topics who presented talks at numerous conferences. L. Prince who coauthored the first article proposing the term “microemulsion”,^24^ included in the first chapters of his early book^16^ a partially fair discussion about some disagreements regarding the use of this term. This early text is worth reading, particularly because it was relatively simple with much less experimental techniques than recent works. The book from D. Langevin^3^ presents a review of concepts and advanced experimental techniques, as well as an overview of current models’ alternatives. It is worth noting that this remarkable text clearly mentions that there are ongoing debates regarding the use of the term “microemulsion” and the nature of the mesophase structures.

In the present review, we address the misleading use of the term “microemulsion” and the multiple meanings of the word “curvature” depending on the adjective used to modify it. We present a simple model for the structure of SOW systems at the so-called optimum formulation, where highly specific and useful properties can be found in both equilibrium and dynamic situations, such as in emulsions.

While useful, the references cited in this review represent only a small fraction of the vast literature on this subject in review books and articles on the basic knowledge and know-how from original contributions to critical advances. This does not imply that all the not-cited publications are unsatisfactory but that there is an overwhelming volume of published material and that the purpose of this review is not a full review of literature work that has been divulgated. Instead, this review focuses on presenting the basic behavior of simple SOW systems with sufficient information to understand how certain inappropriate nomenclature has contributed to confusion in the field.

## Basic Phase Behavior and Occurrences in a SOW System at Equilibrium According to Winsor’s Most Simple Model

Oil and water are incompatible liquids that cannot be mixed into a single phase, and this situation can be represented by the Hansen solubility parameters (HSP) that numerically indicate the difference in polarity of two substances.^25,26^ The addition of a third component type, i.e., an amphiphilic structure with two different parts (and thus a double affinity), does not allow using the HSP approach. Surfactants, referred to as C by the Winsor original model,^27^ are molecules that at least contain a polar group called “head” with some hydrophilic affinity to a water phase (called *A*_CW_) and a nonpolar group, generally a hydrocarbon called “tail” that has a lipophilic affinity for the oil phase (called *A*_CO_). Consequently, surfactants may be soluble in water because of their headgroup, in oil because of their tail, or in both when the two affinities are more or less balanced.

Accordingly, surfactants can best satisfy their double affinity positioned at the interface, and thus the surfactant interfacial adsorption is the crucial feature. When the oil–water interfacial area is fully covered, the extra surfactant separates or, in most cases, goes to the phase having the higher affinity for it.

However, since the surfactant always has a part that does not present a good affinity for the oil or water phase, the usual solubility as single molecules has often a (low) molecular concentration limit, which is in general related to temperature as the minimum Kraft point for ionic species or the cloud point for nonionic ones.^6^ For instance, above the Kraft point, the ionic surfactant either precipitates or solubilizes under the form of aggregates, known as micelle in water with typically 50–100 molecules and whose radius is about the size of the amphiphile straight or bent tail, around 1–2 nm. The same effect happens with the excess surfactant in oil with the occurrence of the so-called inverse micelle structure that is often smaller. This aggregation tends to remove the incompatible part of the surfactant from the solubilizing phase, at a concentration called the critical micelle concentration (CMC), extensively described in the literature, in particular in tutorial books or booklets.^6,28^ The CMC is generally a small concentration but very dependent on the surfactant structure, not only the tail length but also the headgroup. For instance, it is about 0.008 mol/L in water for a hydrophilic ionic surfactant like sodium dodecyl sulfate. However, it is much lower, around 0.00006 mol/L, for another water-soluble specie like dodecyl pentaethoxylate, because of the importance of head structure on aggregation. Its decimal logarithm decreases almost linearly with the number of carbon atoms in an alkyl tail and also increases linearly as the ethylene oxide number of an ethoxylated nonionic increases.^6,29^ The CMC is also very sensitive to external effects of solubilization in solution, like the salinity and hardness of the aqueous phase and the temperature.

In the presence of both oil and water in the system, the surfactant molecule has a preference in its partitioning and is mostly in one of them. This occurs usually as a single phase solution of micelles or inverse micelles, with the other (excess) phase containing very low residual surfactant concentration at or below the corresponding CMC.^30^

In a system containing surfactant in both water and oil, the phase behavior is determined by the surfactant affinity balance for one of the phases. Since the affinity is the negative of the chemical potential, it has to do with the molecular interactions, attractive or repulsive. Winsor^31^ proposed the ratio of affinities *R* = *A*_CO_/*A*_CW_ as the numerical criterion, with an exact balance of affinities of the surfactant in oil and water when *R* = 1. It was a single term criterion like the empirical HLB value of the surfactant^6^ or the surfactant critical packing parameter CPP at the interface,^32^ but many studies indicated that the interactions at interfaces also involved different other terms to be estimated, such as the oil nature, the brine salinity, the temperature, and the pressure. In the early 1970s, the commercial influence of enhanced oil recovery (EOR) led petroleum companies and government/university R&D institutes to rediscover Winsor’s previous work. They started to consider the equivalence of the interaction ratio *R* = *A*_CO_/*A*_CW_ = 1 to a zero chemical potential difference written as the so-called surfactant affinity difference SAD = *A*_CO_ – *A*_CW_ = 0.^33^

The calculation of the ratio or difference in affinity is complex even in very simple cases, in particular because of the many different phenomena and variables involved. Also, the different assumptions concerning the interactions play a role, sometimes in disagreements in some theoretical or experimental approaches. It can be said that the considerable published knowledge, even now, does not allow one to accurately calculate a value of the chemical potential ratio or difference and that the corresponding variables to be used in the theoretical aspects are not easy to handle and measure. Winsor’s proposal was the first suggestion using a set of several practical formulation variables, while the other approaches are dealing only with one descriptor of the system’s behavior.

The first representative parameter was called the surfactant hydrophilic–lipophilic balance (HLB)^34^ and represented the proportion of the surfactant having a preferred affinity for water, stronger than for oil, i.e., corresponding to a negative SAD value. It was a very good name, but it was only calculated with an approximate relation like the 1/5 of the wt % of the poly(ethylene oxide) group for alcohol ethoxylates. The other proposal known as the surfactant critical packing parameter (CPP)^23^ was an extension of the very early wedge theory^35^ that used a representation of a conic association plug of surfactant molecules with water and oil aggregated into a micelle, reverse micelle, bilayer vesicle, or multilayer flat or curved liquid crystal, and the so-called “microemulsion”. This last name was a new nomenclature that produced a confusion because these systems, particularly the bicontinuous type that does not contain real independent oil or water small drops but domains with arbitrary shape and curvature significance, logically labeled “crazy mixed-up stuff” by Scriven in a chapter of his book.^21^ One of the confusions was also using the same curvature meaning for the micellar surfactant aggregation (1–2 nm size) and an actual emulsion liquid drop (1000 or 10,000 times larger) for which the wedge theory cone model cannot have logically the same influence, as mentioned by the author Harkins in his original publication.^35^

In the 1970s, the reported practical variables in a crude oil reservoir were very easy to understand and measure in a first simple equivalent model. They were the water phase nature (salinity of the brine S), the oil nature ACN (i.e., the number of carbon atoms in a *n*-alkane) (or its equivalent EACN with the same behavior), and the temperature and the surfactant nature (e.g., its head and its tail).^36,37^ Some other variables were known to be important candidates to participate in describing specifications of a petroleum reservoir system, such as pressure (downhole), and the composition (water/oil ratio WOR, which is not necessarily unity) and surfactant concentration Cs, as well as alcohol type and concentration, if present. Thus, it is evident that even in a very simplified case, there were 8 variables, and thus using a single variable criterion like in HLB or CPP is obviously not enough.

**Figure 1:**
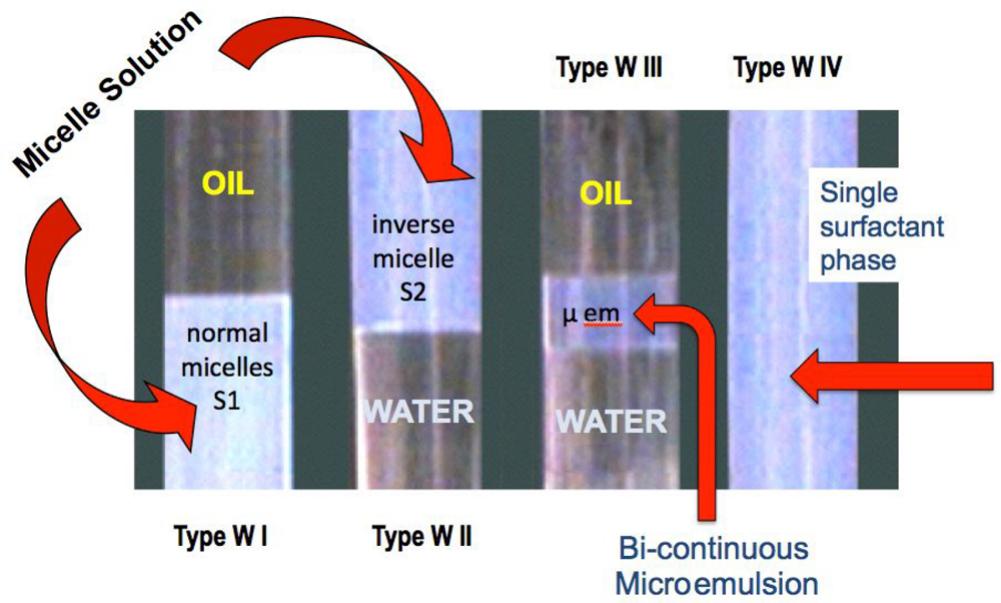
Four different types of phase behavior of a SOW system according to Winsor type I, type II, type III, and type IV. The system eventually contains an alcohol cosurfactant and is at a proper temperature that avoids surfactant precipitation.

This was a puzzling situation in practice, and it is important to understand how the problem was dealt with, according to the high number of different formulation variables but with a simple effect resulting only in the 4 different cases of SOW systems’ phase behavior, as seen in Figure 1, first proposed in the Winsor initial series^27^ and improved in his book^31^ and the invited review in the first volume of chemical reviews.^38^

Figure 1 indicates the 4 types of phase behavior in a surfactant-oil-water system at a concentration higher than the CMC, for instance 1–2 wt %. On the left are the two most common cases when the surfactant (with eventually an additional cosurfactant to avoid precipitation such as a short alcohol) goes mostly to the water phase (type Winsor I with S1 normal micelles water solution) or to the oil phase (type Winsor II with S2 inverse micelles oil solution). The excess phases are completely transparent, with a very low surfactant concentration below the CMC.

The bluish color indicates the phase that contains most of the surfactant because dispersed structures are present, which result in natural light side- or backscattering, like the usual blue of the sky. The surfactant aggregate size, micelles, or other structure may vary and produce more or less opacity depending on the case. In the present review, a proper situation is selected to attain easy visibility of the phase containing the surfactant through a slight Tyndall effect, as noted by Hoar and Schulman^39^ with the presence of ordinary S1 micelles or S2 inverse micelles originally called “oleopathic hydromicelles”.

In Winsor type WIII phase behavior, most of the surfactant is in a so-called middle phase in equilibrium with two excess phases (oil and water).

## Use of the Name “Microemulsion” Can Lead to Confusion in Practice

The WIII type looks as the intermediate between type WI and type WII, and is a very specific case as far as the formulation is concerned, but it does not necessarily indicate a clear middle phase structure characteristic. This phase is now generally called “bicontinuous microemulsion” since Schulman proposed the term “microemulsion”^24^ and Scriven added the “bicontinuous” adjective to satisfy the found evidence.^40^ However, even if it appears to be between WI and WII types, this WIII middle phase cannot be logically represented as a mixture of micelles and inverse micelles, as proposed recently,^41^ since these aggregates in WI and WII cases have different external solution phases.

In type IV phase behavior, the percentage of the phase containing the surfactant has increased to the whole volume. It could be because there is more surfactant in the system or because there is more solubilization of the excess water or/and oil in the phase containing surfactant in any type case, in particular in the WIII type.

It is not known whether the middle phase is a water or oil solution and how much it contains of both and how, and this is probably why Schulman and colleagues^42^ used at first the term “transparent oil-water dispersion stabilized with soap and alcohol” that corresponded to the evidence at their time.

Winsor’s 1948 original studies were meant to determine the phase behavior change when there is a variation in one formulation variable that alters the ratio R of interactions of the surfactant with oil and water or its surfactant affinity difference SAD.

**Figure 2:**
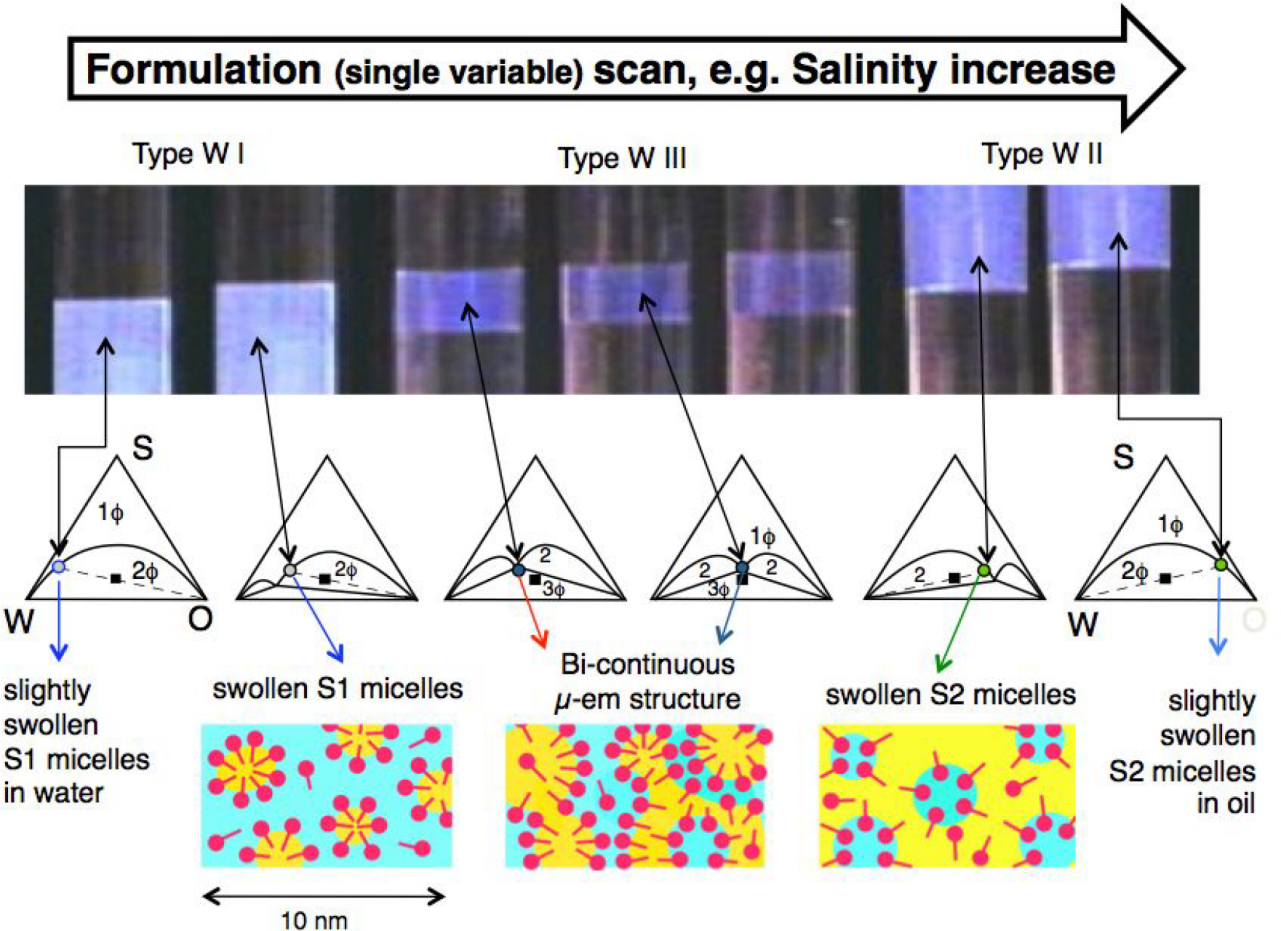
Formulation scan in a SOW system, increasing the water salinity from left to right with all other variables constant (SAD and HLD increases from left to right).

Figure 2 shows the phase behavior of a surfactant-oil-water system with a composition represented at the square point in the SOW ternary diagrams (about 10–15% surfactant and at unitary water/oil ratio). The surfactant and oil nature are constant as well as the temperature and other conditions, except for the salinity of the aqueous phase, which is increasing from left to right, in what is called a continuous formulation scan, here a scan of the water salinity. The effect of increasing this variable is to reduce the interaction of the surfactant headgroup with water (Acw), and thus the R ratio is higher, or to augment the surfactant affinity for the oil, increasing SAD, and corresponding to the same trend for the hydrophilic–lipophilic deviation (HLD = SAD/*RT*^43,44^).

It is seen that in the middle of the scan, there is a salinity range in which a WIII three-phase behavior is obtained in the test tubes. This corresponds to a Winsor SOW diagram with a triangular three-phase zone (3ϕ) surrounded by three biphasic zones (indicated as 2ϕ), the extremely narrow one just below the 3ϕ triangle not being shown. This kind of representation has been published in many places, and the reader not familiar with it can check the literature.^45−47^

The picture in Figure 2 indicates information that will be used later, i.e., that the surfactant phase in WIII cases has a much smaller volume than in the lateral WI and WII cases. Thus, it contains a higher concentration of surfactant, whatever the solubilization structure. It also indicates that the light scattering is less, and this is important data since it certainly indicates different aggregated arrangements in WIII surfactant middle phase, which has not been commented on as far as the authors know. The point is that this diminishing light scattering cannot indicate a smaller micellar size since the solubilization of oil and water in swollen micelles increases from type WI to WIII and from type WII to WIII.

The test tubes scan also indicates the solubilization variation in the surfactant phase in the type I and type II zones, according to the remaining volume of the excess phases. The schematic drawings below the diagram indicate the usually proposed structure of the single phase containing most of the surfactant, i.e., swollen micelles in type I and type II zones and a so-called bicontinuous microemulsion structure in the type III zone.

Figure 2 also indicates that for a unit water/oil ratio (WOR), the salinity formulation corresponding to the center of the WIII zone has the lowest 1ϕ point (blue circle), i.e., the highest solubilization and the lowest interfacial tension according to Chun Huh relation,^48^ and his calculation with a simple flat structure model.^49^

**Figure 3:**
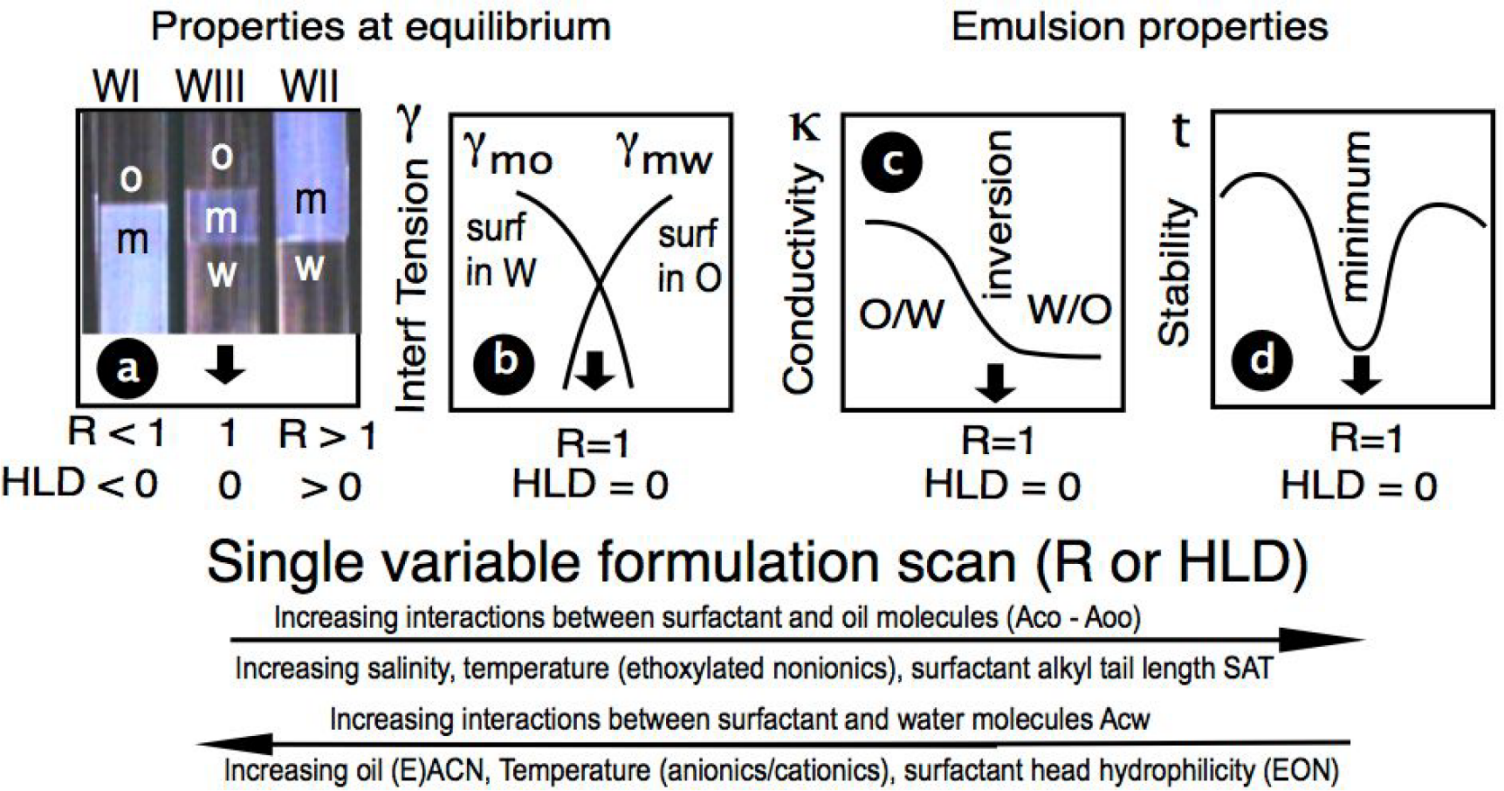
Summary of the variations of surfactant-oil-water (equilibrated and emulsified) system properties along a formulation scan. The arrows indicate the optimum formulation, when variables’ effects on the interactions of the surfactant molecules with oil and water are equal.

This central value of the WIII zone in the formulation scan is the so-called optimum formulation for enhanced oil recovery because it corresponds to the minimum tension as seen in Figure 3, and thus has the best recovery found in the late 1960s with surfactants.^50^ It is also called the optimum formulation for emulsion breaking in processes such as crude oil dehydration^51−54^ because of the corresponding emulsion stability minimum shown in Figure 3.

This trend of phase behavior change (WI > WIII > WII or inverse) with a continuous variation of a formulation variable was found to be qualitatively general by Winsor and the following researchers, with any of the variables susceptible to alter one interaction of the surfactant with oil and water (S, ACN, T, P, SAT, EON...), and thus R, SAD, or HLD.^29,43,55−57^

Figure 3 also shows the variation of two emulsion properties that have a very specific interest in practice, i.e., the emulsion type (indicated by the electrical conductivity) and the emulsion stability (indicated as the time required to separate a certain proportion of one of the phases of the system, for instance 70% of the oil phase). The direction of variation of the HLD formulation expression is indicated in the lower part, according to the definition.^1,2^

However, many cases are more complex than the presented example in the previous Figure 2 and should be appropriately interpreted with some equivalence for practical applications like mixture of surfactants, oils, and brines, the presence of cosurfactants, the influence of the composition, etc. Over the past 45 years, numerous publications have addressed these complex situations. However, to remain at a basic understanding level, they will not be addressed here.

**Figure 4:**
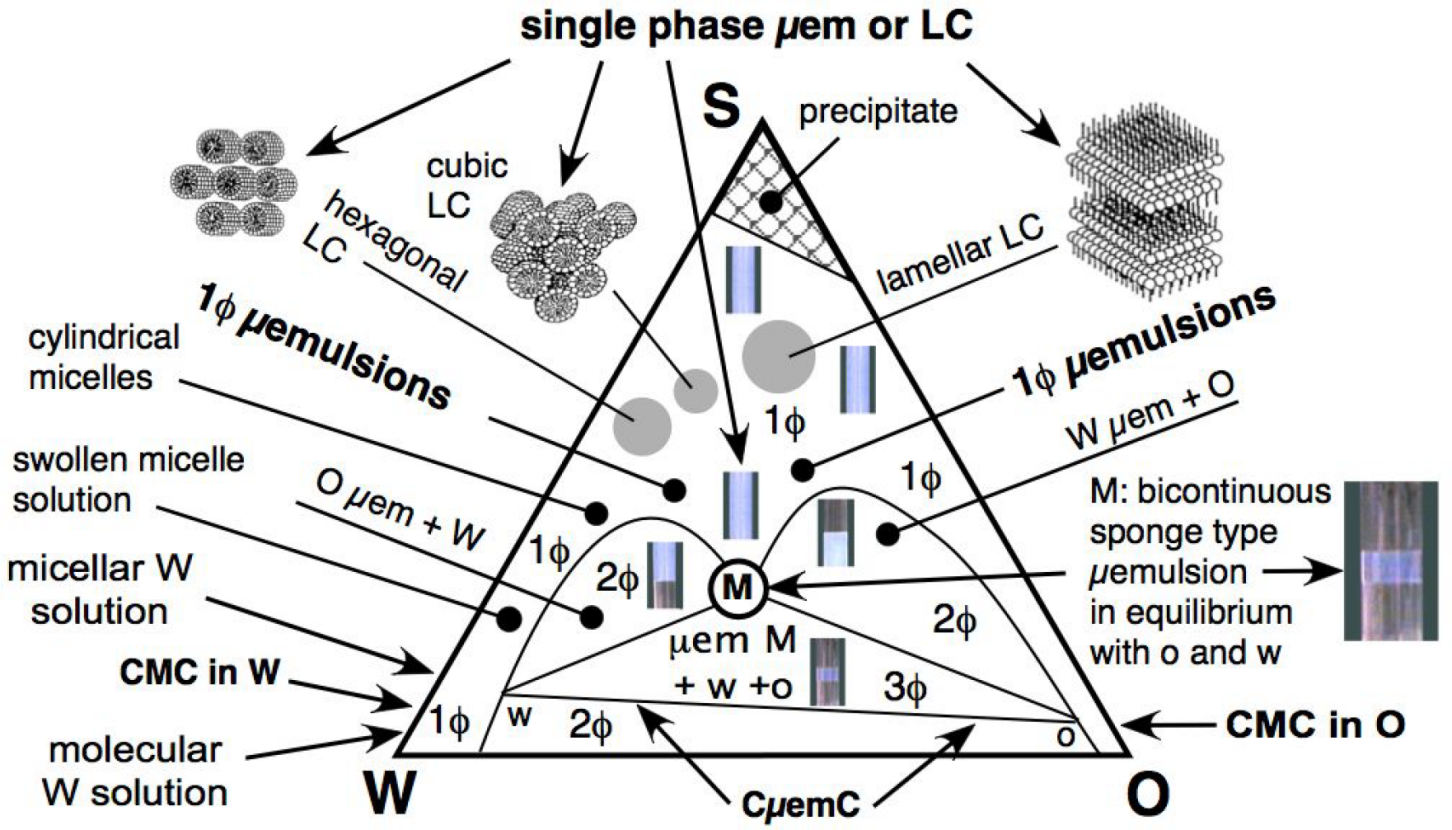
Winsor III typical diagram updated with the information accumulated in the past 40 years about the structural characteristics of the phase containing the surfactant, and misleadingly called microemulsion, as if it simply were an emulsion with microdrops.

Figure 4 shows more information in the most complex WIII type diagram at different compositions, not only at low surfactant concentration and unit WOR at the square point in Figure 2. Figure 4 indicates the influence of the surfactant concentration (Sc) on the actual phase behavior. At high Sc there is a single phase zone (1ϕ), and it is not only in the WIII case shown. This happens in all diagram types seen in Figure 2, and thus it can be said that a Winsor IV single-phase behavior (see Figure 1) takes place in all cases at high surfactant concentration. Nevertheless, the observation of a single phase system in the upper part of the diagrams does not give the proper information about the diagram type nor the molecular arrangement mesostructure, which must be determined by specific methods.^58,59^

The second issue to be considered with Figure 4 is that a large enough range of WOR variation in a WIII diagram produces the same type of transition as the formulation variation around the optimum. For instance, a change from right to left or the opposite, lower than point M (or slightly higher than M), produces a scan WI > WIII > WII or WI > WIV > WII, depending on the surfactant concentration.

The third issue is that the aggregation structure to solubilize the surfactant molecules at high surfactant concentration may be quite different from a spherical micelle with a 1–2 nm radius. It happens not only for pure selected systems but also in many practical cases. For instance, in a crude oil/brine system, asphaltene molecules are very lipophilic surfactants that associate first at very low concentrations in 3–4 molecules small micellelike aggregates or as multimicelles much bigger, known as clusters, when their concentration is higher. How much higher depends on the maltenes nature (e.g., paraffinic, aromatic, with resins or naphthenic acids), temperature, and pressure.^60−63^ This variation of aggregate size and nature may alter the interpretation of some measurements, particularly for the size, shape, and curvature meaning of existing mesostructures.

Figure 4 represents a combination summary of numerous studies for many pure nonionic surfactant systems in the past 50 years by renown researchers such as Shinoda and Kunieda,^64^ and later Kahlweit and Strey,^65^ with similarity with previous studies carried out by people from industry research on practical cases like EOR,^66−70^ which were published in completely different kinds of conferences and journals, and with a different approach, in particular the selected scanned variable. Additional confusion came from arbitrarily changing the previously used logical terminology in SOW systems, calling the water A, the oil B, the surfactant concentration γ, etc.^65^

In most theoretical studies, the temperature is generally the only formulation variable to be used. This has some advantages, such as the possibility of increasing or decreasing it without making new systems, but also some inconveniences such as the range limits, and the fact that temperature alters essentially all interactions, thus many different effects are to be discussed. On the contrary, the salinity only changes the interaction of water with the surfactant head,^68^ or the ethoxylation effect for the nonionic surfactants head (EON).^71^ The oil ACN, which was the first to be historically used for a formulation scan in the University of Texas group,^72^ compared with the interaction with the surfactant alkyl tail length SAT, and resulted in a general equation for all cases,^73^ is thus probably the simplest theoretical formulation scan. Nevertheless, it must be noted that the results indicated in Figure 4 are quite general for a WIII case and apply to most variable scans and are thus consistent with the many theoretical and practical publications.

Micelles in W or inversed micelles in O are located on the left and right sides of the diagram at a low surfactant concentration, but above the CMC. Their shape is mainly spherical, exchanging surfactant molecules in permanence with the saturated molecular solution. When more surfactant is added, the number of micelles increases. For a pure surfactant, there is essentially no change in the micellar arrangement, but in some cases, other types of structured arrangements can be formed and/or coexist. When the representative point enters inside the diagram, i.e., in a zone with both water and oil content, the micelles solubilize the lower content phase inside and are then called swollen micelles because their size increases (as seen in the drawings in Figure 2). This phenomenon corresponds to the so-called solubilization, since it means some apparent amount of oil is inside the micelles in the water solution or some water is inside the inverse micelles in the oil solution.

When the quantity of solubilized phase incorporated inside the micelle becomes too large, an excess of this phase appears in some zones of Figure 4. There is a two-phase behavior, i.e., a swollen micellar phase that is saturated (and sometimes misleadingly called microemulsion) and an excess phase. It is indicated as O μem + W in the left 2ϕ zone and W μem + O in the right 2ϕ zone.

Below these two diphasic zones (2ϕ), there is a triphasic triangular zone (3ϕ) in the WIII diagram that separates in a “central” surfactant-rich middle phase microemulsion (M) having an aggregation mesostructure, and two O and W excess phases. Above these three multiphase zones (2ϕ and 3ϕ), there is a large single-phase zone close to the upper boundaries of the two-phase zones, which has also been called a microemulsion. Nevertheless, it is essentially known that it does not contain ultrasmall oil and water droplets, as proposed by Schulman in 1959,^24^ or both micelles and inverse micelles, as suggested by Acosta et al.^74^ This latter publication introduced the same curvature term for micelles and drops, when a “micellar curvature” or “microcurvature” term (used later on here) would be clearer, even if it is still misleading because there is no interface as in a real emulsion. Consequently, it can be said that the structure of this middle phase M, and the single phase found above it, is still uncertain and depends on the interpretations of results with different instrumental techniques or methods described in many publications. The best review with many collaborators^75^ indicated weak and strong arrangements with more or less rigidity or flexibility with different liquid crystal (LC) and microemulsions labels.^76^ In the single-phase zone called 1ϕ, microemulsion above M and bimodal curves, the actual structural changing of WOR or surfactant concentration will eventually stay homogeneous with some variation in light scattering but not up to the typical whitish aspect of classical emulsions.

The conductivity variation from left to right in the Figure 4 diagram in the single-phase zone (1ϕ), from almost pure water (SW side) to pure oil (SO side), seems to indicate a smooth variation, and it could be seen as percolated micelles and inverse micelles or merged micelles and merged inverse micelles into what Scriven has called a bicontinuous structure^77^ or microstructure.^78^ Other studies have indicated that liquid crystal structures with more or less flexibility, depending on the amount of oil or water inside, can occur with a more or less organized arrangement with hexagonal, cubic, or lamellar shapes.^79^ Such structure can have a network of slightly organized surfactant molecule zones like cylinders or wormlike cylinders, cubic arrangement, lamella, bent layers or bilayers, vesicles, and completely disorganized shape domains of almost pure water and oil zones, but not with spherical shape.

It can be said that there is a continuous transition in the upper part of the (1ϕ) zone, sometimes with a precipitate formed as rigid crystal structures at very high surfactant concentration, as indicated in Figure 4. When more oil and water is added, moving downward in the triangular zone, there are flexible liquid crystalline globular domains, whose size decreases with dilution or becomes looser and more elongated by percolation, thus resulting in a bicontinuous microemulsion. This can be described simply as organized surfactant molecules, simple or multiple films, surrounding more or less elongated excess oil and water phase globules.

## Emulsions and Microemulsions Have Quite Different Microstructures

In microscopic studies performed in the past century, the drops of the emulsions were mainly spherical (or only slightly deformed when subjected to shear or dilation) in a size of several micrometers. Mechanical stirring is usually required to produce an emulsion from a SOW equilibrated system at some surfactant concentration. Energy is required in emulsification because it is linked to the increase in surface area with the γΔA term, that is significant unless the tension is extremely low, as it happens close to optimum formulation. Spontaneous changes tend to reduce the surface area and thus to coalesce separated drops, making an ordinary emulsion not thermodynamically stable, even if the breaking time scale can be very long.

Emulsions in SOW systems contain a water phase with high electrical conductivity (in particular if it contains an electrolyte or salt) and an oil phase that generally is not conducting. Thus, depending on the type of external phase, the conductivity is high or low. Microemulsions show smooth conductivity variation in a formulation or WOR scan. In contrast, emulsions exhibit a marked change in conductivity around the inversion point, for instance, when it changes from O/W to W/O, at the so-called phase inversion temperature (PIT)^80,81^ in scans performed with ethoxylated surfactants systems with the temperature as the formulation variable. In numerous Shinoda’s publications, there was no citation of Winsor’s work published 15 years before, nor the name microemulsion proposed by Schulman 10 years before. The only other study mentioned by Shinoda’s group was the Griffin HLB single variable concept.^82^ As far as the phase behavior was concerned, it was shown not in a Winsor ternary diagram but in other bidimensional diagrams, including the formulation variable temperature and the composition WOR,^83^ as will be discussed later.

The microemulsion name and its relationship with other variables such as salinity or surfactant type as in EOR were only published in the 1980s, i.e., 30 years after Winsor’s work, and just 2 years before Kahlweit and Strey’s group started to extensively enter this scientific area insisting on the scanning with temperature. This situation is noted here because comparing different approaches probably started only at this time, but still with temperature as the most used formulation variable in theoretical studies.

As discussed in detail above, the microemulsion was a confusing nomenclature incorporating the “emulsion” term, because it is a single-phase system, indicated (1ϕ) in Figures 2 and 4, i.e., with no interface at equilibrium and thermodynamically invariable. In other words, it can be said that the only spontaneous changes are provided by the Brownian motion, which takes place at the molecular level, i.e., around a very few nanometers of thermal fluctuation.

At this size level, the only curved object is the micelle or inverse micelle found in type I or type II surfactant phases, with a shape close to a sphere. But micelles are water or oil soluble and have thus no real interface and do not coalesce, unlike emulsion droplets that are typically much larger. Moreover, micelles are stable because they avoid the precipitation of the surfactant. Additionally, micelles are always exchanging surfactant molecules all the time and very fast.

In a different situation, emulsion drops are in the 1–10 μm size with internal pressure higher than the external fluid as indicated by the Δ*P* = 2γ/*R* equation, where γ is the interfacial tension and *R* the radius of the sphere. When two drops are bumping, the small one has a superior pressure and thus coalesces sooner or later into the larger one. Consequently, the drops tend to become larger until they are all coalesced in a single phase. This does not happen with micelles because two bumping ones do not coalesce to make a bigger single one. There is no such pressure variation from the inside to the outside of a micelle, the interfacial tension is not measured at the micelle surrounding but at an interface. Micelles just can appear or disappear depending on the surfactant molecules' permanent quick exchange with the solvent/external phase.

**Figure 5:**
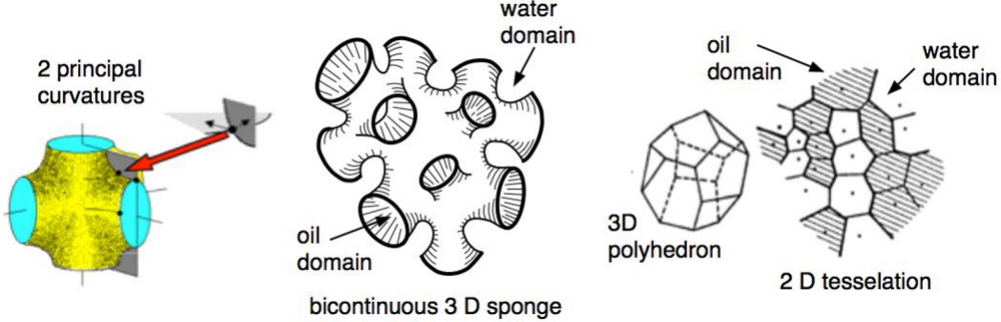
Bicontinuous shapes percolated-like and more or less merged micelles and inverse micelles, sometimes called mixture of wormlike elongated structures, as bicontinuous sponge shape, or randomly interdispersed tessellations shown here in 2D and 3D space.

As indicated in Figure 4, the actual objects in the monophase zone (1ϕ) can be far from spherical. A situation that can also be deduced from the names proposed by Talmon or Zemb such as “interdispersed domains tessellation” or “disordered open connected cylinders” in studies linked with light, X-ray, and neutron scattering.^84,85^ The deformation from sphere is due to the presence of oil and water globules with other shapes, sometimes variable from place to place as in quasi-crystal structures filling the whole volume with multifaceted domains.^86^ In such cases, the word curvature that is simple to understand for spherical drops or spherical micelles is confusing to apply with stable thermotropic or unstable lyotropic liquid crystals or microemulsion globular domains that are more or less merged, as shown in Figure 5.^87^

A book containing amazing drawings concerning the curvature of strange surfaces with saddle shape aspect has been published by Hyde^79^ as well as a review article on mesophases that the reader should check if the book is too long.^88^ This clear article is enough to understand the confusion on curvature concepts for dispersed globular objects found in bicontinuous microemulsion and sometimes called a sponge structure. It is worth noting that, in general, these structures are not containing exactly repetitive shape domains, in particular with a zero mean curvature splay saddle shape, aspects already mentioned in Scriven’s original article^77^ with their corresponding patterns^89^ and relation with low interfacial tension.^90^

Other terms such as “surfactantless microemulsions” have been confusedly used for systems where the so-called Ouzo effect occurs.^91,92^ It happens in certain mediterranean aperitifs like the Greek ouzo and the French pastis containing a large amount of a low molecular weight amphiphile, e.g., 40–45% of ethanol, only about 1–2% of a terpenoid oil very insoluble in water like anethole, and slightly more than 50% water. These anis-flavored liquors are clear but become cloudy when excess water is added. The mechanisms behind the effect are not fully understood in particular because the oil phase is in a very small amount, not at WOR close to unity as in typical microemulsions in the center of a ternary diagram.^93,94^ Eventual aggregates of molecules can create structures or a solution with no structure at all that allows for the formation of a continuous, transparent phase.^93^ The whitening of the ouzo or pastis drinks when water is added may be just a solubility limit when the ethanol content goes below 30%. These systems are often referred to as surfactantless microemulsions,^95−97^ although this can be misleading because they differ significantly from traditional microemulsions formation,^98^ thus adding even more confusion to the terminology.

## Curvature Confusion with Both Emulsions and Microemulsions

The name curvature has been used in emulsion systems with a typically spherical interface in type I and type II cases with drops of essentially pure oil or water liquids of about 1–10 μm, eventually in a wider range in scales. In all cases, the natural light scattering is very strong and the emulsions are very opaque. Schulman selected the name microemulsion because of the known trend that much smaller spherical objects exhibit very little light scattering,^24^ particularly when smaller than 1/10 of the wavelength, e.g., 0.05 μm (50 nm), for the sunlight. However, objects that are not spherical, particularly percolated micelles or wormlike or cylindrical globules, can produce inaccuracies and confusion about the curvature term meaning.

**Figure 6:**
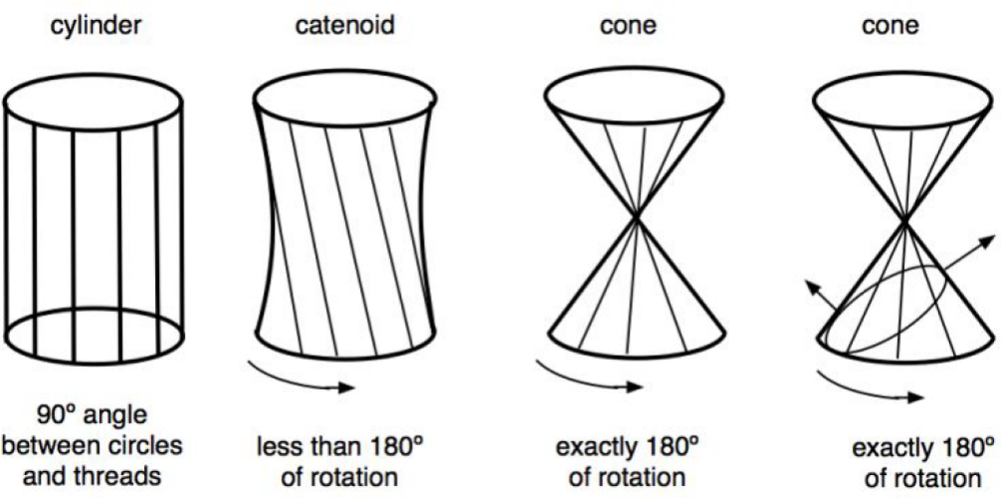
Not very evident concept of the curvatures and mean curvature for a nonspherical shape. The right figure indicates that the principal curvatures are not the most obvious ones, with the two cuts by planes.

There are several issues producing inconsistencies with the same term: (i) Understanding what is a curvature of a line in a bidimensional space is simple because there is no alternative to the center of curvature and the bending concept. In three dimensions, it is not the case for a surface shape very different from a sphere because the immediate feeling is that there are variable curvatures, depending on how the surface is cut by a plane to produce different 2D curved lines. The name “mean” curvature requires at least two curvatures, i.e., two cuts of the surface, which are somehow arbitrary, mostly taken as coming from the more curved and less curved 2D cuts, a choice that is not evident. (ii) Looking at Figure 6, it is seen that a cylinder can be provided when attaching threads perpendicular to two solid circles and that two significant curvatures are provided in this case, by the circle (horizontal cut) and by any vertical cut that results in straight lines that form the surface. These are the so-called principal curvatures that result from perpendicular cuts. Suppose one of the circles is rotated with respect to the other. In this case, the surface is still produced by straight lines forming a so-called catenoid, with one of them producing the lowest surface area, i.e., the shape of a liquid interfacial film between the two circles. On the other hand, when the rotation of a circle is exactly half a full turn (180°), the surface is still made with pure straight lines with a double cone shape. For this case, the most logical understanding of the surface curvature is from the circle size and the distance between the circles. However, these are not principal curvatures obtained by putting a vector perpendicular to the surface and rotating a plane around the vector. One of the curvatures (the lowest one) will be zero along a straight line and the other is an oval or elliptic cut, which does not seem very significant or the simplest.

Moreover, the problem of curvature significance is unlikely when the surface is the interface of two immiscible fluids phases. For instance, for a cylindrical liquid stream, the two different curvatures are not happening but only temporarily. As a faucet is opened an almost cylindrical stream of water is produced with a diameter that decreases because of the gravity. It then quickly becomes a series of droplets, with the radius depending on several parameters, and showing a strange result in which the surface area increases because of the work produced by gravity motion.

The curvature has importance on the difference of pressure on the two sides of an interface. The typical rising and curving of the interface at the vertical wetted wall or a thin capillary cylinder clearly indicates the meaning of the Δ*P* = ρgh = 2γ/*R* equation at the interface. When two drops are bumping, the difference in pressure inside them produces the small one emptying in the large one by different mechanisms. When two micelles are bumping, this does not happen. This is probably because the micellar solution has no interface and no difference in pressure between the inside and outside. In fact, a micelle does not empty into another bigger one, i.e., they do not coalesce. This is because micelles are surfactant aggregates, and what is in motion are the molecules exchanging with the external solution or displacing their position in the arrangement structure if there are not spherical aggregates. Therefore, a microemulsion does not change with time but at the molecular level keeping the same average balance of interactions.

In a normal drop size, any shear will produce elongation, thus the interfacial area and the pressure difference in the drop will reestablish the spherical shape spontaneously when the shear disappears. This will be a much shorter variation than the interdrop film thinning as in the Marangoni effect elasticity.^99,100^

The second confusion concerns the association of the transparency-opacity with the microemulsion name and domain size, spherical or different from it. For spherical objects in the micrometers range, i.e., close to the light wavelength, the opacity is reduced when the droplets are getting smaller, and this is consistent with the use of the microemulsion name. The issue is that this might be a misperception because if the micelles get more swollen as it happens when the formulation approaches the optimum in type I and type II phase behavior then they become bigger and are more likely to be more whitish (or less transparent) because of light scattering.^101^ However, it was seen in Figures 2 and 3 that this trend does not follow in the WIII zone in which the surfactant-containing middle phase scatters less.

**Figure 7:**
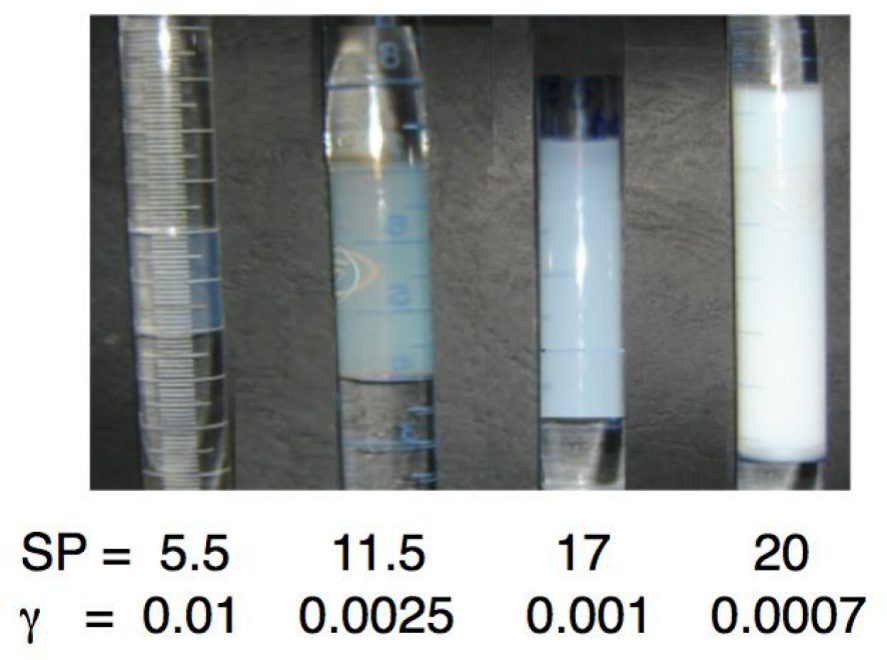
Optimum formulation for different systems but with equal surfactant concentration of 15 wt % in the system and WOR ∼ 1 in the middle phase. Solubility parameter (SP) and interfacial tension γ in (mN/m) at optimum formulations of different scans are shown, with quite different performance.

Nevertheless, for this middle phase microemulsion, the more opaque or whiter it is, the higher the solubilization parameter and the lower the interfacial tensions. This is seen in Figure 7, showing the optimum test tubes in scans with different surfactant-oil-water systems but with the same surfactant concentration.

The interfacial tension value for the fourth (right) case is very difficult to measure and quite approximate (±30%), but the fact that it is more whitish than the third one clearly indicates a lower tension and a higher solubilization (that is very close to 100% of oil and water with only 15 wt % of extended surfactant).

This kind of formulation is quite appropriate for EOR, pharmaceutical creams, dehydration, and other practical cases.

It is worth remembering that when increased performance is produced, as seen in Figure 7, the range of the formulation variable for having a WIII phase behavior is reduced, with the solubilization parameter varying inversely to the HLD range of WIII phase behavior.^102−104^ It thus means that the exact position of the optimum is extremely sensitive to the formulation scan value.

The curvature concept with an usual emulsion is essentially a temporary value, i.e., the inverse of the drop’s radius. Consequently, it will change with time as well as with the energy introduced to make the emulsion, e.g., the stirring apparatus and work input. On the contrary, the curvature in a microemulsion with very small domains is supposed to be a constant independent of the formation story, and of the stirring, whatever the mixing process. However, it depends on the way the curvature is calculated. For a sphere, it is without confusion, but for a domain that is not a sphere, it can be confusing because it depends on the way the curvature is defined as overall, partial, spontaneous, intrinsic, etc., or by a formula which is not *C* = 1/*R* that is the only obvious one in the two-dimension flat space. In one of the well-known examples of the confusion, a so-called characteristic curvature of a surfactant (CC) term is used,^105^ the curvature is defined essentially from the oil/water solubilization at a WOR = 1. It is obtained by calculating the ratio of the oil volume (equal to the volume of water because WOR = 1) to the surfactant volume, that is a layer on top of the spherical oil or water domain.

Vol_oil_ or vol_water_ = 4/3 π*R*^3^ and vol_surf_ = 4π*R*^2^*L*, where *R* is the radius of the oil or water sphere and *L* is the thickness of the surfactant layer.

The ratio, basically the performance of the WIII system with equal oil or water solubilized in spherical domains (wrongly assuming that normal micelles an inverse micelles exist at the same time), is SP = *R*/3*L*, where *R* is the radius of the spherical oil or water domain and *L* is the thickness of the surfactant layer around them.

For *L* = 1 nm, as is the case for dodecyl sulfate and a measurement of solubilization performance SP = 5.5 and γ = 0.01 mN/m, a radius of 15 nm is indicated. If SP = 17 and γ = 0.001 mN/m, a radius of 50 nm is obtained, which is probably as whitish as would be expected from a 0.2 μm nanoemulsion. Thus, the opacity of the emulsion or microemulsion is not very unique information.

**Figure 8:**
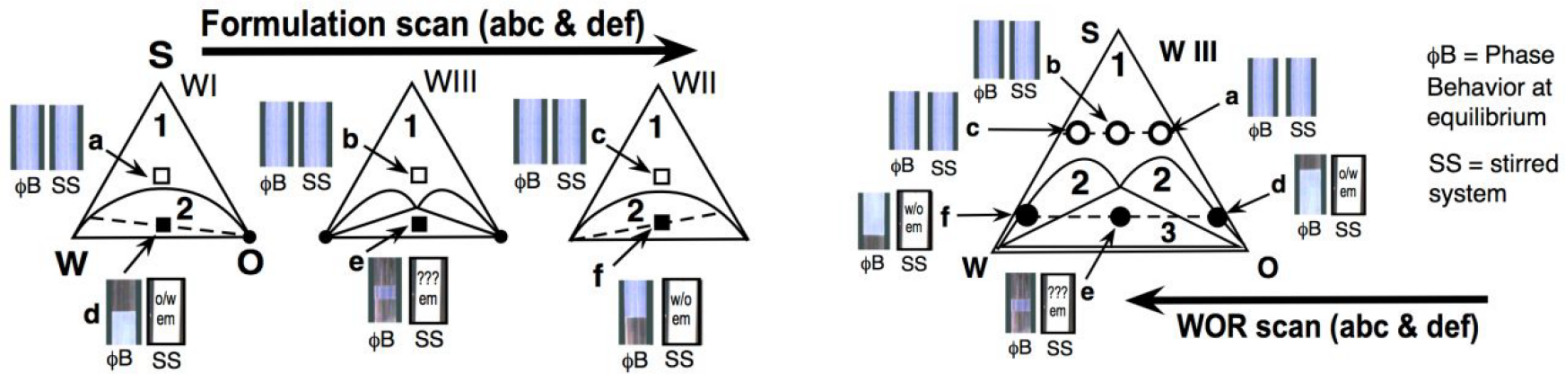
Changes in the phase behavior (ϕB) aspect of the equilibrium system and stirred system (SS) to emulsify in formulation scan or WOR scan that alters the surfactant affinity balance for oil and water phases.

Figure 8 indicates what happens when a SOW system from different Winsor types is stirred because of a formulation scan (left) or a WOR scan in a WIII diagram (right).

At each indicated point (a to f) of the triangular SOW diagrams, there are two representations of test tubes, the left (ϕB) being the aspect of the equilibrated system and the right one (SS) being the aspect of a just stirred system, i.e., the emulsified case. At the square or circular white points indicating the composition-formulation of systems a, b, and c, the equilibrated and recently stirred systems exhibit the same aspect, because there are single phases with objects too small to result in a whitish color. Note, however, that if the M point is not far away from the base of the triangle, a single phase happens at a low surfactant concentration. But in any case, the bluish color will be the same for the equilibrated and stirred systems.

On the contrary, in the black points d, e, and f in the polyphasic cases types I, II, or III, there are 2 or 3 phases, resulting in the formation of emulsions with a whitish color that are much whiter than the equilibrated surfactant microemulsions, whatever the surfactant is in oil, water, or middle phase.

**Figure 9:**
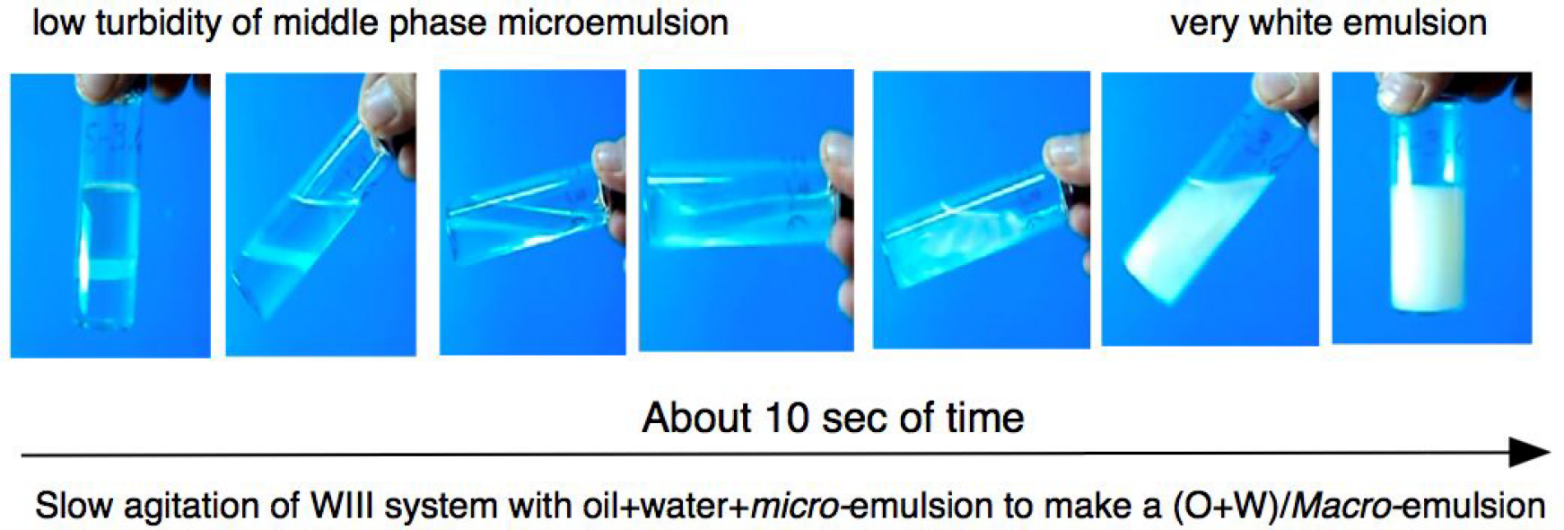
Very slow and weak stirring of a WIII system at equilibrium that results in a three phase emulsion which is very white, i.e., having small O and W micron-sized droplets in the M external phase, but, according to the light scattering in the initial picture, much larger droplets than the swollen micelles or other mesostructures in M.^106^

Figure 9 indicates how a slow agitation of a SOW system at equilibrium at point “e”, in the lower part of the three-phase triangle of a WIII type in the previous figure, produces a mini- or macroemulsion. The characteristics of the process are (1) the system is at the optimum formulation of the scan, and thus at the minimum interfacial tension (a low one of about 0.005 mN/m, according to the solubilization parameter). (2) Even if the agitation is very low and slow, a whitish mini- or macroemulsion is produced with small O and W droplets in a continuous phase which was the middle phase microemulsion M, as soon as the small shearing produces some stirring. After less than a minute, excess phase separations, i.e., an oil phase and a water phase, appear at the top and lower part of the tube, respectively. The return to the original aspect, i.e., the complete phase separation of both excess phases, requires only 4–5 min because the (macro) emulsions are very unstable at optimum formulation.

## Conductivity with Some Variation versus Formulation or WOR Changes in Emulsions and Microemulsions

Information on the conductivity is easy to handle with an emulsion with drops of one phase dispersed in the other continuous phase. Emulsion conductivity is measured under stirring, thus homogeneity to avoid separation of different phases is attained because of different densities, and the inversion is easy to detect with very quick conductivity change over a very narrow range in the scan. Shinoda’s temperature scan fairly showed this phenomenon in nonionic surfactant system at the optimum formulation called the PIT.^107^

In the a-b-c scans in the previous Figure 8 right diagram, the microemulsion conductivity varies from low to high, but smoothly and continuously, with a much lesser jump than in the d-e-f change.

Later Shinoda and Kunieda^108^ found that the WIII case at equilibrium was essentially at the same formulation as the PIT but that it was theoretically something different. Thus, they called it HLB-temperature as far as phase behavior was concerned.

They did not say it, but they probably found out that a WIII formulation was attained and that they were two cases, one in the 1ϕ zone and one in the 3ϕ zone. When they were stirred, the aspect of the first one (1ϕ slight turbidity blue color) was kept, while the 3ϕ system was becoming extremely white as seen in Figures 8 (in SS test tubes) and 9, i.e., it becomes a real emulsion.

## Effect of Microemulsion Structure in the Low Stability of WIII Emulsified Systems

**Figure 10:**
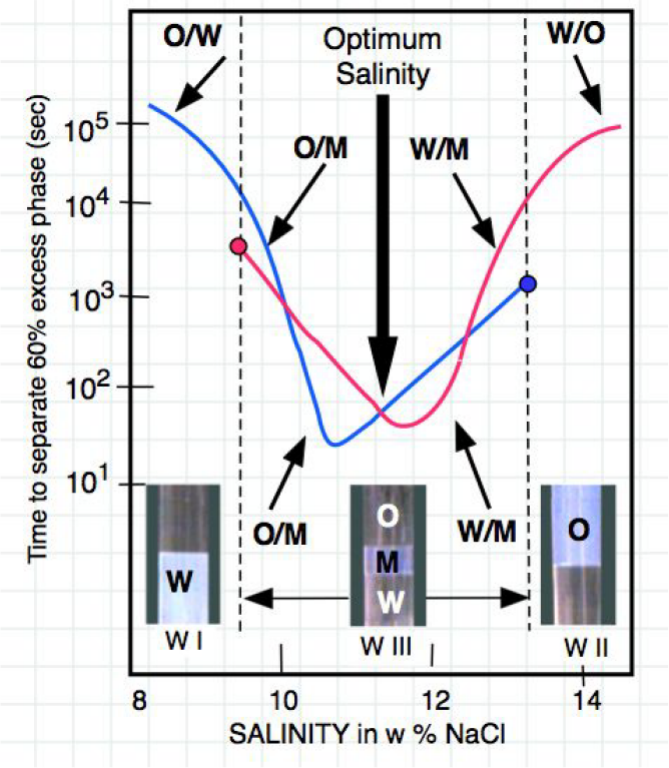
Stability versus salinity scan for 2 phase emulsions, i.e., O/M and W/M, in the three-phase system zone with 2.5 wt % SDS surfactant and WOR = 1.

As shown in Figure 10, stirring a SOW system with 2 or 3 phases produces an emulsion if the surfactant concentration is lower than the M point. When the WOR is not far away from unity then the external phase is the one containing the surfactant as it was known to be through the so-called Bancroft’s rule.

In a WIII case, the three-phase emulsion consists in drops of excess water and excess oil in an external phase that is the middle phase M. Thus, there are two emulsions, i.e., W/M and O/M, and they break quickly at the same time but often one quicker than the other depending on the formulation with respect to the optimum, as shown in the 1980s.^109,110^ This is indicated as a sum up in Figure 10, showing the stability of the O/M and W/M emulsions where M is the middle phase and the others the excess ones. If the three phases are left, both the two excess phases are separated at the same time very similarly as in both O/M and W/M cases.

Therefore, with a three-phase emulsion, the stability is measured as the velocity of separation of excess phases, excess oil in the upper part and excess water in the lower part. However, observing the separation of oil in an O/M emulsion or the water in the W/M indicates they are essentially the same with both O and W drops elongated when moving in the remaining emulsified center zone.

At salinity below the value corresponding to WIII zone, i.e., lower than *S* = 9.5%, the phase behavior is WI and the two-phase emulsion is O/W. Above this value, the emulsion becomes O/M, and its minimum is at a salinity slightly lower (*S* = 10.5) than the center of the WIII range *S* = 11.3%, which is indicated as the optimum by a black arrow. On the other hand, at a salinity above the upper limit of the WIII zone (over *S* = 13.5%), there is a WII phase behavior and a W/O emulsion that becomes a W/M emulsion in the triphasic zone. It is also seen that the minimum stability of the W/M emulsion is found at *S* = 11.8%, slightly above the optimum formulation.

Thus, the optimum formulation where the interfacial tension between excess O and excess W is minimum, is found at the crossing point of the stability curves of the O/M and W/M emulsions. Therefore, if a mixture of the three phases is stirred, the formed emulsion separates both oil and water and the figure curves can be drawn as in Figure 10.

It is worth noting that with very low interfacial tension systems, the WIII range is not very wide, much less than the current case where the minimum γ is 0.01 mN/m. It must be said also that in practice, with a lower tension minimum, it could be much wider, with a quite different formulation for the minimum stability. In other words, in a three-phase emulsion, the excess water could be separated faster than the excess oil or the contrary, and this is why both stability curves rise again after passing by a minimum somewhere inside the WIII zone.

It is interesting to try to understand why the emulsion stability is always much lower in the WIII zone, whatever the case. The following discussion is to explain that this is due to the bicontinuous structure of the middle phase microemulsion M, i.e., changes at the boundary between two and three-phase formulation.

**Figure 11:**
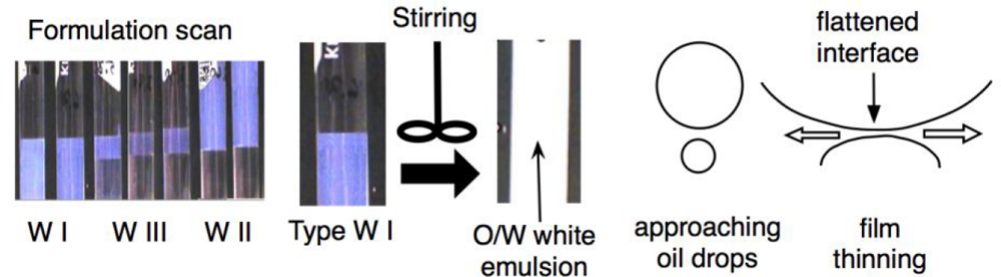
Stirring of an equilibrated type WI system to make an emulsion and starting of emulsion breaking due to the interdrop film thinning.

Figure 11 indicates the formation of emulsions by stirring an equilibrated SOW system in the WI case that is very close to the boundaries with WIII, for instance, between the second and third tubes in the salinity scan in the left picture.

The stirring can be carried out with the WIII case by taking only the excess oil phase and the M phase, so there is always an excess oil phase with practically no surfactant (i.e., below CMC) and an aqueous phase that contains most of the surfactant. In the WI case, the water phase contains solubilized oil inside swollen micelles, and in the WIII case, higher solubilization happens in some structures, such as elongated micelles or more complex aggregates.

As indicated before, in the WIII range the volume of the middle phase is much less than in the surfactant containing water in the very close WI case, with the same amount of surfactant. This means that the surfactant concentration in the middle phase M is much higher than in the water phase in WI, typically 5 times in the present case. This occurrence produces more micelles but also allows the micelles to touch and probably to form other structures as elongated cylinder or “worm-like micelles” by percolation or fusioning. It is logically expected that at higher surfactant concentration, there will be more micelles and thus more light scattering, but the evidence is the opposite, as already seen in Figure 2 picture. Consequently, it could be thought that the radius of the worm micelles (like a twisted cylindrical structure) is smaller than the spherical micelles and thus has a higher curvature and less scattering.

The fact is that in the WIII case, the interfacial tension is lower than in the WI case and this also favors the elongation, since the related energy term, i.e., the product of the tension by the area (γA), is reduced.

**Figure 12:**
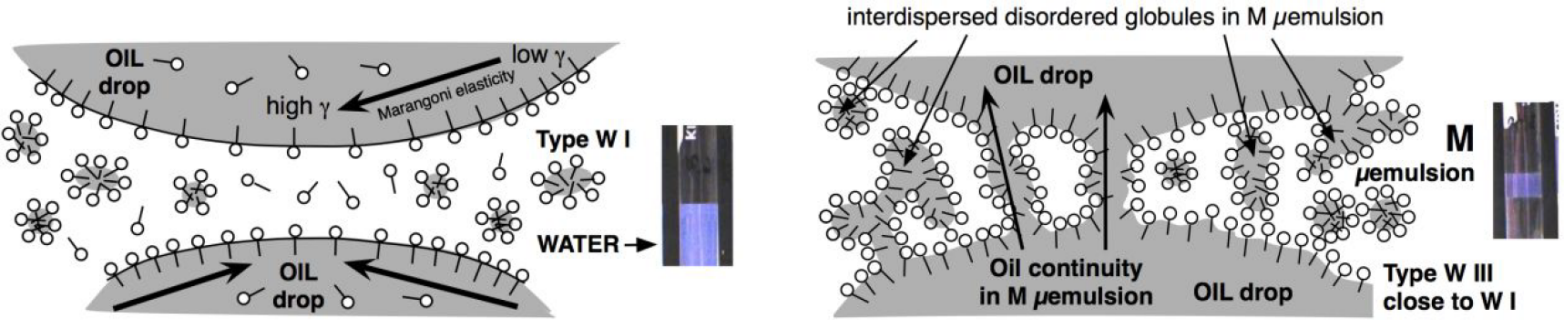
Situation when two big oil drops approach with the formation of a film of water external phase that can resist thinning (as in WI type formulation in the left figure) or very quickly breaks with a middle phase M film containing elongated aggregates (as in the WIII case close to the WI boundary in the right figure).

Figure 12 indicates these differences between the WI and WIII types, even if the formulation variation is very small. The change is just due to a considerable variation of the surfactant concentration and interfacial tension low enough to increase the surface area with a minimum energy input like the Brownian motion, which has a consequence only at the molecular level, i.e., 1–2 nm, not at a drop size level that is 100–1000 times larger.

Figure 12 indicates the change in structure in the surfactant-containing phase close to the WI–WIII boundary. In the WI case (left), the water contains a low concentration of micelles, and the typical Marangoni elasticity tends to spontaneously limit the surfactant phase film thinning because of the tension gradient. In the WIII case (right), there is much more surfactant available in the external phase and thus in the film, often with elongated aggregates (like nonspherical micelles), so that the adsorption at the interface is faster, and the tension gradient disappears. Moreover, the lower tension close to optimum is another reason to eliminate the tension gradient and the elasticity of the film. A similar difference will happen close to the WIII–WII boundary inverting the oil and water roles.

Another interesting note is that with a bicontinuous structure shape in the film structure, even temporary, some short bypass can be readily available, as seen in the right figure. Thus, coalescence becomes instantaneous because of the difference in pressure between the drops of different sizes. The amount of bypass probably depends on the exact position in the WIII zone, and thus justifies the minimum of both emulsion (O/M or W/M) cases slightly displacing one from the other as seen in Figure 10.

**Figure 13:**
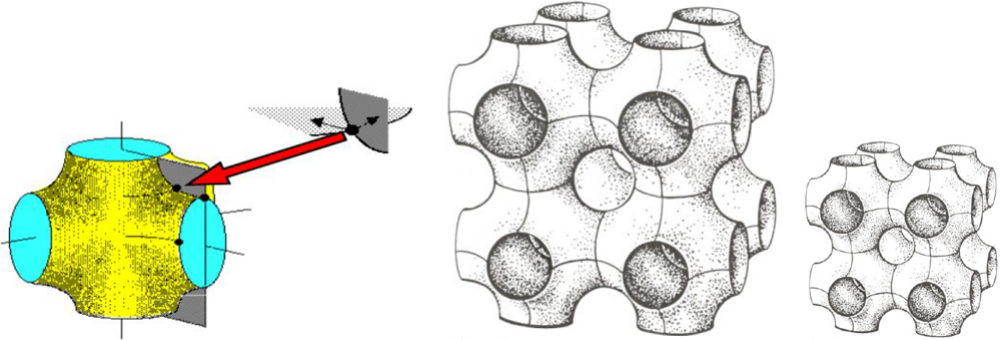
Bicontinuous surface shape with fusioned spheres in three directions with a zero or almost zero mean curvature domain shape (left). Globular domain size is twice as large in the center figure than in the right one, but with a higher solubilization and lower interfacial tension, because the film thickness *L* is the same whatever the size of the individual globule.

Figure 13 indicates that in the simple bicontinuous case (with similar size globules as in a perfectly symmetrical Schwartz structure), the volume of a single domain depends on the third power of the globular size, proportional to *R*^3^ if it were a sphere. In contrast, the surface area depends only on the second power *R*^2^ of the globule size and on the thickness of the surfactant layer *L*, i.e., a characteristic length like the surfactant extension that varies with the tail. Comparing this structure at different sizes, the relation between the unit volume and unit surface is the same, but if the surface is covered with a surfactant of characteristic length *L*, which is the same in both globule sizes, the solubilization is not the same but depends on the size of the unit globule, as is evident with a structure made by cubes, cylinders, or spheres.

Consequently, even with the same structure with a zero main curvature, the bigger size unity with lower C_1_ and C_2_ principal curvatures in the center drawing produces a higher solubilization performance SP and as well has a lower interfacial tension at optimum formulation as discussed elsewhere.^78^ This means that the mean curvature is not a unique criterion to characterize the situation, at optimum formulation when it is zero in all cases, whatever the surfactant, oil, water, and temperature values. It will be the same with varying the globular sizes with a distribution of domain shape and size.

Thus, the surfactant characteristic curvature name CC proposed some time ago^105^ is not a surfactant characteristic at all, and the performance index PERFIND is probably more useful^104^ to make comparisons. Moreover, it is not a curvature nor a microcurvature either because such a concept requires an oil/water interface or at least a solvent to dilute a surfactant as micelles or other aggregates, and the drops or micelle size depends on other parameters than the surfactant descriptor, in particular on the temperature, and water salinity including different ions.^111^

## A 4-Dimensional Spatio-Temporal Microemulsion Model Could Be Simple to Understand Structural Shape Complexities Happening Close to the Optimum Formulation of SOW Systems

This last section is dedicated to propose a 4-dimensional (4D) spatiotemporal model of SOW simple systems at or close to optimum formulation according to the information available. This model will make it relatively easy to explain in tutorial activities without the usual puzzling confusions, as it is found in many detailed reviews, even recent ones with a lack of clarity in the microemulsion and curvature terminology.^112^

The model consists in using, as the only mechanical energy generation, the Brownian motion thermal fluctuations, i.e. the bumping of the surfactant structures by water or oil molecules about 10^22^ times per second. In accordance with the experimental data following the small particles displacement by microscope, the average Brownian motion is arbitrary and quite reduced in energy, thus likely to have an effect only at the molecular level, like spherical micelles or other surfactant aggregate structures. It means that it happens at the scale of a very few nanometers, not at the usual macro-emulsion drop size, which is typically millions of times bigger in volume or mass. Consequently, with a very high number of Brownian bumps, the actual result will be an average effect in time and in space of changes at the 1–10 nm scale.

**Figure 14:**
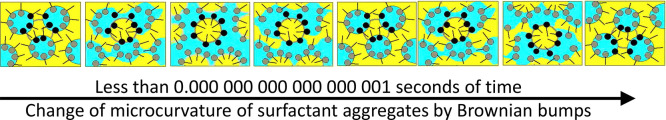
Rapid change of the spherical micellar aggregation with time in a case very close to optimum with Brownian motion altering the position of the surfactant molecules in the micellar-like arrangement fluctuation. In general, at optimum formulation the aggregates will be nonspherical, very flexible at very low tension, and more or less percolated, but the principle will be similar to this scheme that explains time and space bicontinuity.^113^

Figure 14 indicates the arrangement of surfactant aggregates in a SOW system at or close to optimum formulation, shown here as spherical micellar size happening at very low surfactant concentration, but that could be occurring similarly with nonspherical aggregates, as mentioned previously. The Brownian bumping of the surfactant molecules in these aggregates can slightly change their position and can result in a change in the local “microcurvature” of the aggregate, thus changing the average from micelle to inverse micelle for some time, and occurring in longer times than the bump, but still over an extremely short time. These micelles can be more or less partially opened^85^ or incomplete, with no clear understanding on how to account for the curvature in the opened or connected part. Consequently, the microcurvature can become positive or negative from place to place, zero if no separation surface, and during a very short time. Both kinds of micelles at the same time, as proposed in Acosta’s model,^105^ are not possible because both external phases would be required. Therefore, in this case, it is the surfactant film located at the oil–water limit that is practically the external phase. As has been noted previously, the structures can be elongated, in particular because of the low tension close to optimum, and open in a percolated worm type of association by partial fusion of neighboring micelles.

The series of pictures in Figure 14 indicates that this quickly changing microemulsion structure presents a continuity of both the oil and water phase not only in space, but also in time, thus explaining the continuous conductivity variation from type I to type II. This double property would be even more evident in a 3-dimensional case.^113^

As a consequence, making a difference with the names and phenomena happening at the different size levels is not only logical but also necessary to reduce the confusion.

For conventional emulsions, say with a radius of about 10 μm (or in the extended 1–100 μm range), drops are produced from an equilibrated SOW system by mechanical stirring, resulting in a strong light scattering as in the whitish milk. When the relative affinity of the surfactant for the oil and water is changed by a formulation or WOR variation (i.e., passing from a type WI zone to a type WII, or inversely) according to the Winsor multivariable concept, the emulsion (kept stirred) becomes inverted as was corroborated by the PIT name given by Shinoda group in Japan.^16^ Other researchers in France have published interesting data.^114,115^

The supposed intermediate complex situation between the WI and WII cases, more or less understood as a very unstable mixture of the O/W and W/O emulsions, has been described by a catastrophe theory occurrence with a so-called “butterfly” manifold, with double cusp bifurcation, as explained in detail elsewhere.^116,117^

**Figure 15:**
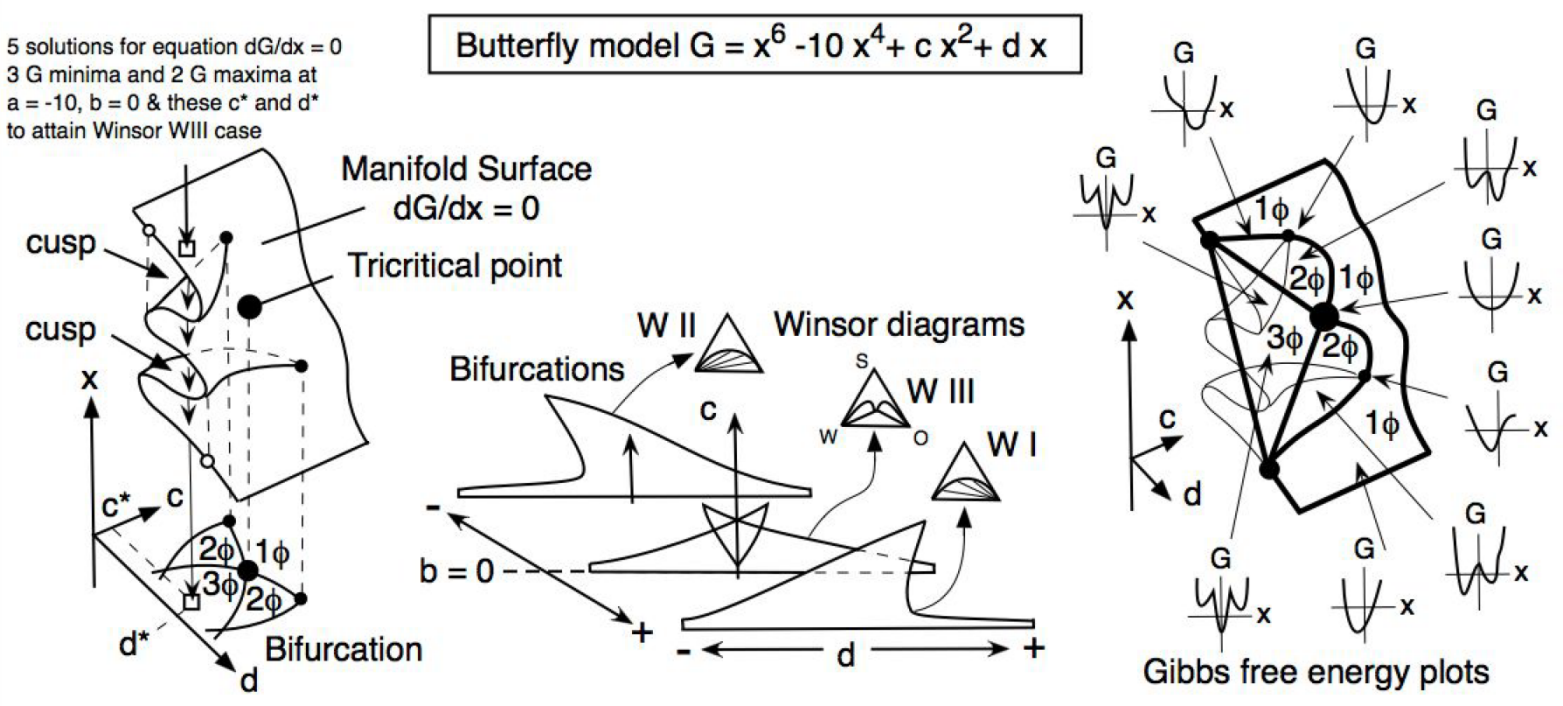
(Left) The manifold and bifurcation aspects in catastrophe theory for a Gibbs free energy *G* model of the 6th order, i.e., with up to 5 solutions to d*G*/d*x* = 0, including 3 minima and thus three phases at equilibrium.^118^ (Center) Several sections at different formulations (*b*) with different surfactant concentrations (*c*) and WOR (*d*) comparing bifurcation plots and Winsor triangles. (Right) Cut out of multiphase zone cusps with a scheme of the free energy plots in a Winsor III type situation. The (*a*) negative coefficient is what results in a double cusp manifold, and thus a three-phase separation at optimum (when *b* = 0).

Figure 15 shows the different sections of this model with a potential of the sixth power. That is the simplest for Gibbs free energy (*G*_abcd_(*x*) = *x*^6^ + *ax*^4^ + *bx*^3^+ *cx*^2^ + *dx*). It has up to three minima, according to the ball in the hole analogy in the right plot *G*(*x*) graphs and thus with the possibility of up to 3 phase behavior. This *G* expression has 4 independent coefficients *a*, *b*, *c*, and *d*, where the “*a*” negative coefficient results in a double cusp indicated in the *c*-*d* map in the left plot, similar to a Winsor triangle diagram.^116^ The effect of “*b*” is the equivalent to the generalized formulation, including *T* and *P* (i.e., HLD from negative to positive). “*c*” is equivalent to the surfactant concentration and *d* to the water–oil ratio.

Phase inversion of (macro)emulsion, like Shinoda’s PIT process, are catastrophe theory changes with time, i.e., dynamic phenomena with high energy by stirring (quick, irreversible, with hysteresis). As far as the phase behavior is concerned, it is an equilibrium situation with no time effect (continuous, instantaneous, and reversible). It does not require external energy, i.e., no stirring with the exception of Brownian motion, i.e., a very low energy input that often requires time to reach equilibrium, unless the formulation is at optimum, i.e., with a very low interfacial tension, even a zero value.

The catastrophe theory model predicted a possible hysteresis for the inversion due to a change in WOR far from WOR = 1, as it is happening in practice.^119^ Exceptional hysteresis can take place for a formulation change,^119,120^ probably because of delay in equilibration.^121^ Catastrophic phase inversion is also used to produce specific solutions, in particular for viscous oil phases.^122^

For the microemulsion misleading name case, different situations happen, and the term microcurvature will be logically used to avoid confusion. The microcurvature is justified by the close to zero mean curvature of the separation surface. However, it is attained as the difference between two very high principal curvatures *C*_1_ and *C*_2_ in a splay saddle structure, which is a very different situation from micelles dispersion happening away from optimum. Also, for a microemulsion to happen, the surfactant concentration must be quite large to get a single phase, whatever the complex structure. It is much higher than the typical concentration of less than 1%, usually used to get a macroemulsion for many applications.

For nanoemulsions,^123^ i.e., very small-size emulsions with interface and instability, it is better to keep them as normal emulsions according to their properties even if the “nano” part is not really appropriate because their droplets are, in general, larger than 50–100 nm,^124^ i.e., larger than the size of a microemulsion domain, which is confusing since nanometer means 10^–9^ m and micrometer 10^–6^ m.^125^

## Conclusions

This review presents arguments sustaining that what is called a microemulsion is not a true emulsion and that the microcurvature is not the same as the usual curvature concept in ordinary (macro)emulsions. Associating the term microcurvature with microemulsions may help to clarify the distinction or may create more confusion if this review’s comments are not properly understood.

A microemulsion does not contains drops but rather surfactant aggregation structures with surfactant-oil-water wedge elements at the molecular level, such as micelles, liquid crystal, or more complex arrangements. Unlike macroemulsions, a microemulsion is a single phase and has no oil–water interface, no pressure difference inside and outside the structures, and is stable over time, not only because of the absence of gravity effect but also because of some kind of mean value with location and time fluctuations. At and close to the so-called optimum formulation, it is bicontinuous and has no internal nor external phase, making it distinct from an emulsion, which is a dispersion of liquid drops in another liquid external phase.

The surface limit between oil and water domains in a microemulsion was said to be a surfactant-rich monolayer or bilayer with a close to zero curvature, i.e., a flat shapelike structure as in some liquid crystals. It was proposed that diluting flat liquid crystals can provide a liquid dissolution. i.e., a single phase with dispersed objects having a zero curvature that can be attained with a shape like a saddle with two principal curvatures, which have different signs, and whose sum is zero. This is a puzzling manipulation of concepts to determine domains similar to almost spherical drops or micelles. According to the current discussions, the domains are of unknown shape, highly variable from place to place at a given time, and along the time at a given place. Thus, the overall microcurvature is a mean or average that could be measured, maybe with a preference slightly positive or slightly negative, even if it is a difference between two very high principal curvatures like in micelle and inverse micelle cases with a small radius.

Using the term “microcurvature” for microemulsion systems could be a way to understand the differences and reduce confusion.

Furthermore, a proper use of the wedge theory and packing parameter with fluctuations when the interfacial tension is very low (at the optimum) will allow one to explain that Brownian motion is enough to provide the necessary fluctuations to have a stable single phase average.
